# Epstein–Barr virus: To be a trigger of autoimmune glial fibrillary acidic protein astrocytopathy?

**DOI:** 10.1111/cns.14336

**Published:** 2023-07-17

**Authors:** Xiao‐Li Li, Jun‐Yan Wang, Liang‐Kang Li, Chun‐Lin Yang, Xue‐Lu Zhao, Bing Yang, Peng Zhang, Bin Liu, Yan‐Bin Li, Zhao‐Xu Zhang, Rui‐Sheng Duan

**Affiliations:** ^1^ Department of Neurology The First Affiliated Hospital of Shandong First Medical University & Shandong Provincial Qianfoshan Hospital Jinan China; ^2^ Department of Neurology, Shandong Provincial Qianfoshan Hospital Cheeloo College of Medicine, Shandong University Jinan China; ^3^ Shandong Institute of Neuroimmunology Jinan China; ^4^ Department of Neurology Peking University People's Hospital Beijing China

**Keywords:** autoimmune glial fibrillary acidic protein astrocytopathy, cognitive dysfunction, enteric glia cells, Epstein–Barr virus, midbrain interpeduncal fossa

## Abstract

**Background:**

Autoimmune glial fibrillary acidic protein (GFAP) astrocytopathy is a novel autoimmune disease of central nervous system (CNS). It is unclear whether Epstein–Barr virus (EBV) is related to autoimmune GFAP astrocytopathy.

**Objective:**

To describe the clinical, laboratory, and imaging characteristics of patients with autoimmune GFAP astrocytopathy.

**Methods:**

The clinical, laboratory, and imaging findings of patients are presented. The levels of GFAP in CSF were detected by ELISA. T and B cell subsets in CSF were detected by flow cytometry. GFAP‐IgG in serum and cerebrospinal fluid (CSF) were tested by cell‐based assay (CBA) and tissue‐based assay (TBA).

**Results:**

All three patients had fever, cognitive dysfunction, limb weakness, and positive GFAP‐IgG with EBV infection in CSF. Enteric glia cells may involve in this disease. Typical imaging findings include the gadolinium enhancement of linear perivascular radial perpendicular to the ventricle, meningeal enhancement (especially in midbrain interpeduncal fossa), longitudinally extensive lesions involving spindle cords, and more T2/Flair‐hyperintense lesions in the periventricular white matter at late stage. The patients had poor response to antiviral treatment and strong response to steroid pulse therapy.

**Conclusion:**

EBV could induce CNS autoimmune response in autoimmune GFAP astrocytopathy. The detection of GFAP‐IgG and EBV may facilitate the early diagnosis in these patients.

## Graphical abstract

Epstein–Barr virus (EBV) could induce central nervous system (CNS) autoimmune response in autoimmune glial fibrillary acidic protein (GFAP) astrocytopathy. The potential pathogenic mechanisms including: directly invade CNS (release inflammatory cytokines IL‐6, IL‐1, et al), CD8^+^ T cell mediated damage, infection‐mediated immune response, and EBV reactivation.

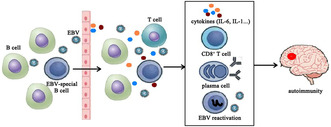

## 1 INTRODUCTION

Autoimmune glial fibrillary acidic protein (GFAP) astrocytopathy, a novel autoimmune disease of central nervous system (CNS), generally involves the meninges, cerebrum, spinal cord, optic nerve, and peripheral nerve with headache, fever, delirium, epilepsy, blurred vision, ataxia, limb weakness, autonomic nerve dysfunction, and so on.^1^

Epstein–Barr virus (EBV) can infect approximately 95% of the world adult population and has been associated with autoimmune diseases.^2^, ^3^ EBV can shuttle between different cell types, mainly B cells and epithelial cells. EBV can switch between a latent and a lytic life cycle. EBV latent infection is found predominantly in memory B cells, but the natural infection cycle and pathological aberrations enable EBV to infect numerous other cell types, including gastric epithelia, B‐, T‐, and NK‐lymphoid cells, astrocytes, and neurons.^3^ The effect of EBV infection on the immune system is thought to contribute to the pathogenesis of autoimmune disease.^3^ So far, it is unclear whether EBV is related to autoimmune GFAP astrocytopathy. Here, we report three cases of autoimmune GFAP astrocytopathy with EBV infection in CNS.

## 2 METHODS

### 2.1 Clinical data

All patients were subjected to detailed clinical examinations, neurophysiological studies, and neuroimaging examinations. Before the procedure and during follow‐up, all the patients were evaluated by a routine brain and spinal cord magnetic resonance imaging (MRI) study at 1.5T (GE signa) or 3.0T (uMR 790) magnetic field strength. The scan include different sequences (T1‐weighted imaging, T2‐weighted imaging, T2‐Flair, enhanced T1, enhanced T2 Flair, T2 STIR). Blood and cerebrospinal fluid (CSF) samples were collected. Ethical approval was granted by the ethics committee of the First Affiliated Hospital of Shandong First Medical University. Written informed consents were obtained from patients before the investigation.

### 2.2 Detection of the levels of GFAP in CSF

CSF of patient 2 and patient 3 at different time points were evaluated for the presence of GFAP. CSF (1:5) samples were added to GFAP‐IgG (immunoglobulin G)‐precoated 96‐well plates. Then 50‐μL horseradish peroxidase (HRP)‐conjugated agent was added to each well except blank control well and incubated for 30 min. After washing five times, 50 μL of Chromogen Solution A and 50 μL of Chromogen Solution B were added to each well and incubate at 37°C for 10 min. The reaction was stopped by sulfuric acid solution. Then the absorbance was measured at 450 nm. All reagents are provided by Wuhan ColorfulGene Biological Technology Co., LTD (Wuhan, China).

### 2.3 Detection of T and B cell subsets among lymphocytes in CSF

CSF was centrifuged, and cell counts were performed. The cell suspension was incubated with APC‐CD3 (Biolegend), Percp‐CD19 (Biolegend), PE‐Cy7‐CD4 (Invitrogen), and FITC‐CD8 (eBioscience) antibodies for 30 min at 4°C. Then, a flow cytometer (Becton Dickinson) was used for detection.

### 2.4 Cell‐based assay (CBA)

HEK‐293 cell lines were transfected with plasmids encoding GFAP homosapiens transcript variant 1 (NM_002055.5). Transfection cells were fixed with 4% paraformaldehyde and permeabilized with 0.2% Triton X‐100 and incubated at 4°C with patients' serum (1:10 dilution), CSF (1:1 dilution), and rabbit GFAP polyclonal antibody (proteintech, 16,825‐1‐AP) (1:500). After washing with phosphate‐buffered saline (PBS), cells were incubated with secondary antibodies (1:200) for 30 min. Then images were captured by fluorescence microscopy.

### 2.5 Tissue‐based assay (TBA)

Rat frozen sections (10 μm) of hippocampus, cerebellar, and small intestine were fixed with acetone for 30 min. Sections were incubated with patients' serum (1:10 dilution) and CSF for 60 min. After washing with PBS, sections were incubated with FITC‐conjugated goat anti‐human IgG antibody (1:200) for 30 min. After washing, slides were labeled with DAPI (4′,6 diamidino‐2‐phenylindole) (Solarbio) and observed by fluorescence microscopy.

## 3 RESULTS

Anti‐nuclear antibodies, anti‐double stranded DNA IgG antibodies, anti‐Smith antibodies, anti‐SSA antibodies, anti‐SSB antibodies, anti‐neutrophil cytoplasmic antibodies, anti‐aquaporin 4 (AQP4) antibodies, and anti‐myelin oligodendrocyte glycoprotein (MOG) antibodies were negative in the serum of three patients. Tests for serum EBV, herpes simplex virus, influenza virus, hepatitis, and mycobacterium tuberculosis were all negative. All patients showed no evidence of tumor.

### 3.1 Case presentation#1

A 33‐year‐old woman initially presented with fever, headache. Gradually, she presented with cognitive dysfunction, dysphoria, and limb weakness. She had no coexisting autoimmune diseases. Reduced muscle strength of bilateral limbs, weakened tendon reflexes of bilateral lower limbs, stiff neck, and bilateral Babinski signs were found in the patient.

**FIGURE 1:**
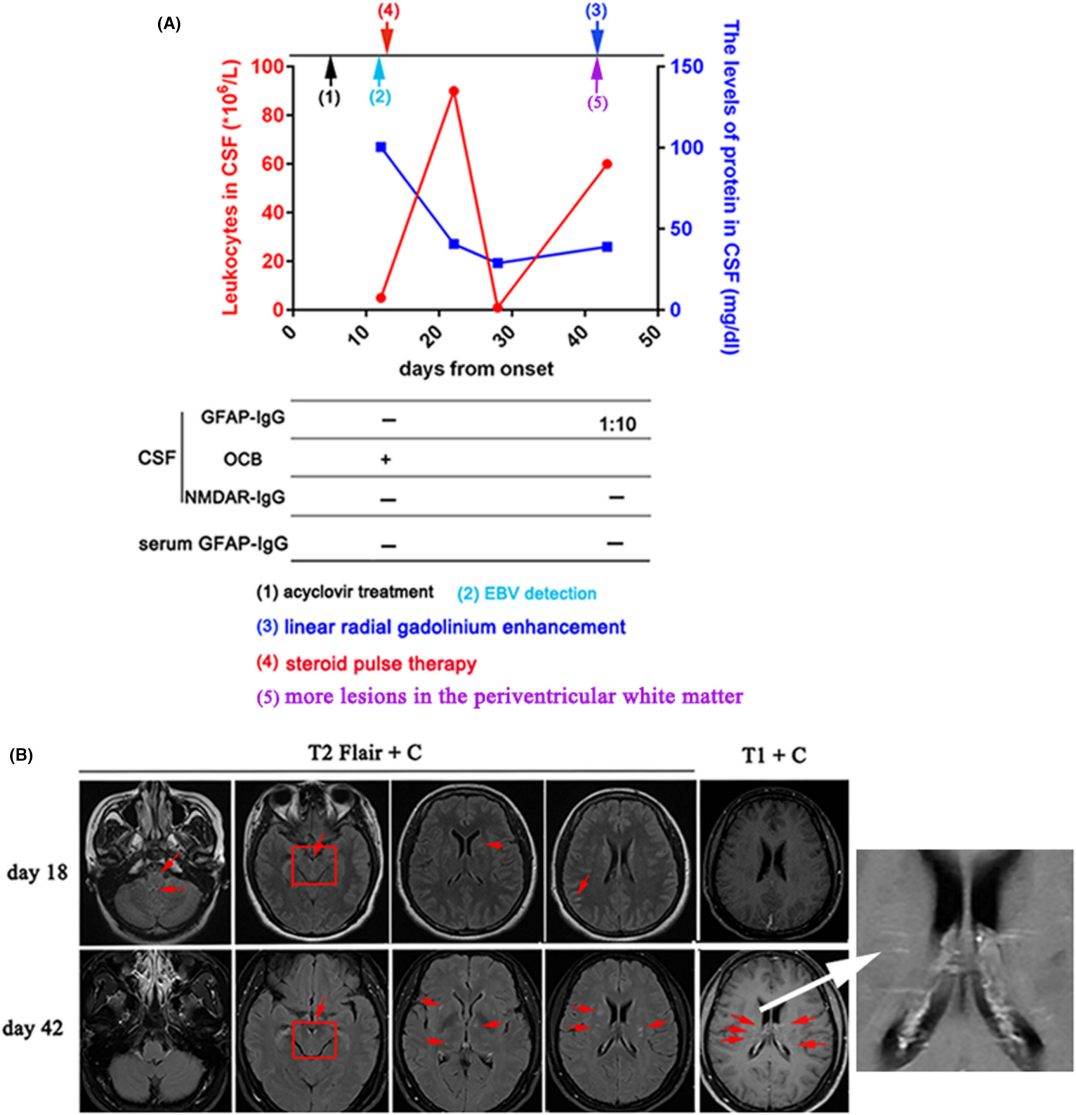
(A) CSF findings during EBV infection and follow‐up. Leukocytes in CSF: red circles and red lines, left y‐axis. The levels of protein in CSF: blue boxes and blue lines, right y‐axis. −, negative; +, positive; OCB, oligoclonal bands. (B) Brain MRI showed obvious meningeal enhancement, including the meninge at midbrain interpeduncal fossa on day 18. Image showed less meningeal enhancement (except for the meninge at midbrain interpeduncal fossa), but linear enhancement oriented radially to the ventricles (white arrow) and more lesions in the periventricular white matter on day 42.

The clinical symptoms had mildly improved after acyclovir treatment. As shown in Figure 1A, CSF analysis revealed normal leukocytes and elevated protein of 100.4 mg/dL on day 12. Metagenomic next‐generation sequencing (mNGS) showed EBV DNA unique reads is two in CSF. GFAP‐IgG was negative in serum and CSF, and N‐methyl‐D‐aspartate receptor (NMDAR)‐IgG was negative in CSF. Oligoclonal bands (OCBs) were positive in CSF and negative in serum. Brain MRI showed obvious meningeal enhancement, including the meninge at midbrain interpeduncal fossa on day 18 (Figure 1B). The clinical symptoms had significantly improved after steroid pulse therapy at 0.5 g/day with tapering doses. Repeated CSF analysis showed high leukocytes (90*10^6^/L) with 70% monocytes and protein of 40.7 mg/dL on day 22, while showed normal leukocytes (1*10^6^/L) and protein of 28.9 mg/dL on day 28 (Figure 1A).

During follow‐up, repeated brain MRI showed less meningeal enhancement (except for the meninge at midbrain interpeduncal fossa), linear enhancement oriented radially to the ventricles, and more lesions in the periventricular white matter on day 42 (Figure 1B). Spinal cord MRI showed no lesions on day 44 (image not shown). Meanwhile, CSF analysis showed elevated leukocytes (60*10^6^/L). Repeated detection of GFAP‐IgG was positive in CSF (titer, 1:10) and negative in serum. NMDAR‐IgG was also negative in CSF. The patient was diagnosed as autoimmune GFAP astrocytopathy and received low dose of corticosteroid and mycophenolate mofetil. The clinical symptoms were significantly improved and no relapse after 3 months of clinical follow‐up.

### 3.2 Case presentation#2

A 60‐year‐old man developed fever, abdominal pain, ventosity, limb shaking, and limb weakness. After 10 days, he presented with cognitive dysfunction and urine retention. He had hypertension and coronary atherosclerotic cardiopathy. Upon neurological examination, he presented with disorientations of place and time, impaired memory, and comprehension. Bilateral Babinski signs were positive.

**FIGURE 2:**
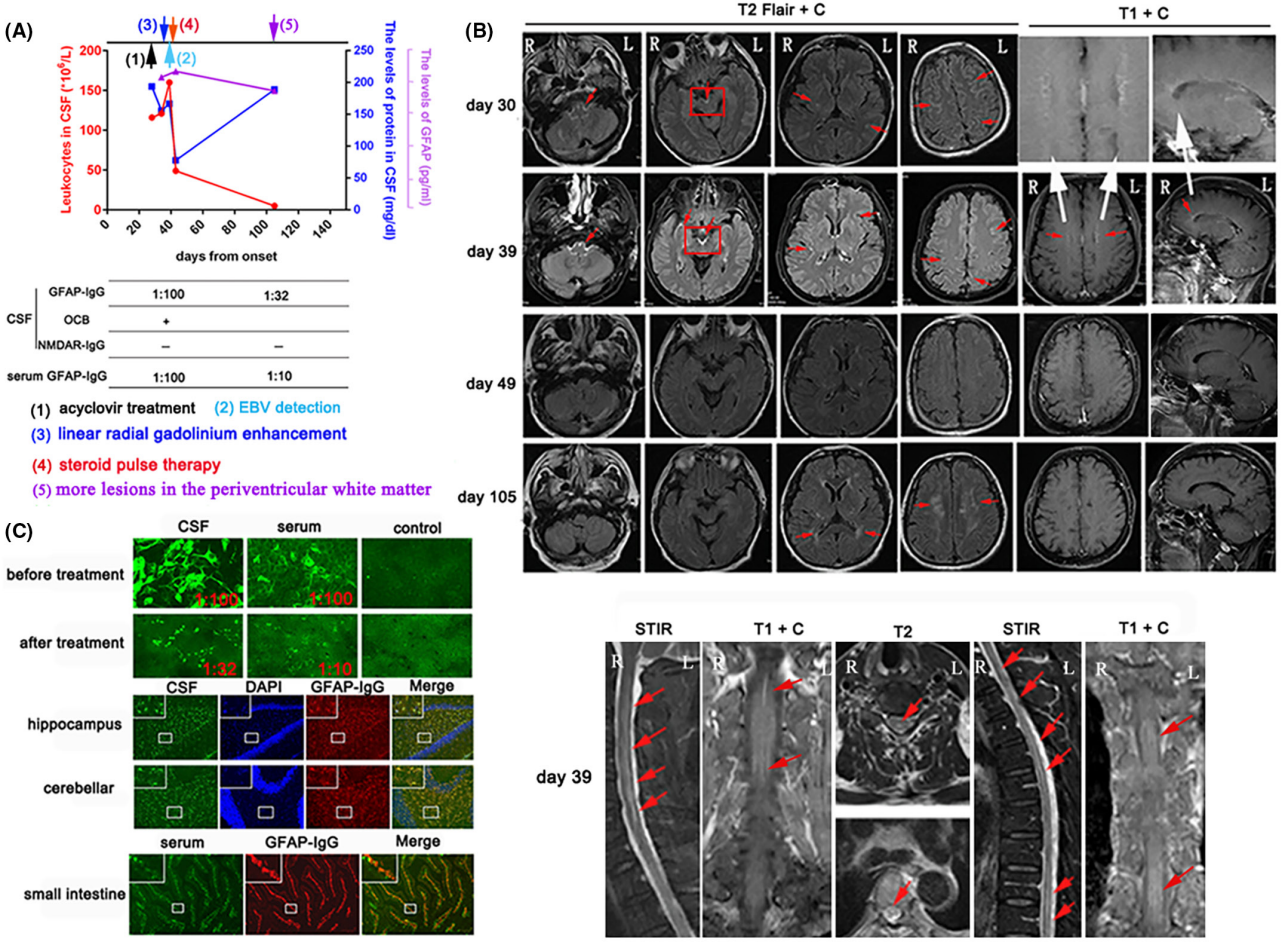
(A) CSF findings during EBV infection and follow‐up. Leukocytes in CSF: red circles and red lines, left y‐axis. The levels of protein in CSF (increased protein may be related to the incorporation of red blood cells into CSF on day 105): blue boxes and blue lines, right y‐axis. The levels of GFAP in CSF: purple triangle and purple lines, right y‐axis. −, negative; +, positive; OCB, oligoclonal bands. (B) Gadolinium enhancement of brain MRI showed meningeal enhancement on day 30. Image showed more serve meningeal enhancement (including the meninge at midbrain interpeduncal fossa) and linear enhancement oriented radially to the ventricles (white arrow) on day 39. Meanwhile, spinal cord MRI showed longitudinally extensive lesions involving the cervical and thoracic spinal cord, with spinal cord central canal and pia enhancement lesions in T1‐weighted images. After hormonotherapy, brain MRI showed no meningeal enhancement on day 49. However, image showed more T2/Flair‐hyperintense lesions in the periventricular white matter on day 105. (C) GFAP‐IgG was detected in the CSF (1:1 dilution) and serum (1:10 dilution) at different times using cell‐based assay (CBA) on day 38 (original magnification ×400). GFAP‐specific IgG in CSF (day 38) of case 2 could colocalized in hippocampus and cerebellar of mice (original magnification ×200). GFAP‐specific IgG in serum (day 38) of case 2 could partly colocalized in small intestine of mice (original magnification ×200).

The clinical symptoms had not improved after acyclovir treatment. CSF analysis showed an increased leukocytes (116*10^6^/L) with 90% monocytes and elevated protein (193.6 mg/dL) (Figure 2A). mNGS showed EBV DNA unique reads is one in CSF. Gadolinium enhancement of brain MRI showed meningeal enhancement on day 30 (Figure 2B). Repeated image showed more serve meningeal enhancement (including the meninge at midbrain interpeduncal fossa) and linear enhancement oriented radially to the ventricles on day 39 (Figure 2B). Spinal cord MRI showed longitudinally extensive lesions involving the cervical and thoracic segments, with central canal and pia enhancement (Figure 2B). GFAP‐IgG were positive in both CSF and serum on day 38 (titer, 1:100, respectively). The CSF of the patient (day 38) in rat hippocampus and cerebella as well as the serum of the patient (day 38) in rat small intestine yielded a GFAP‐IgG‐like immunohistochemical binding pattern (Figure 2C). CSF revealed positive intrathecal IgG OCB. NMDAR‐IgG was negative in CSF.

Based on all the data, autoimmune GFAP astrocytopathy was diagnosed. Subsequently, the patient received intravenous steroid pulse therapy followed with tapering doses. The clinical symptoms were significantly improved. Repeated CSF analysis showed slightly high leukocytes (49*10^6^/L) and protein level of 77.8 mg/dL (Figure 2A). Imaging examination showed no meningeal enhancement on day 49 (Figure 2B). Two months later, CSF analysis showed normal leukocytes. The levels of GFAP and GFAP‐IgG in CSF and serum were significantly reduced (Figure 2A). Lesions of meningitis and spinal cord disappeared (image not shown), while more lesions appeared in the periventricular white matter on day 105 (Figure 2B). The patient received low dose of corticosteroid and mycophenolate mofetil. To date, the clinical symptoms had no relapse after 12 months of clinical follow‐up.

### 3.3 Case presentation#3

A 71‐year‐old man presented with fever, urine retention, occasional ventosity, and loss of appetite. Gradually, he presented with cognitive dysfunction, bilateral hand tremors, and limb weakness. He had hypertension and no coexisting autoimmune diseases. Upon neurological examination, he presented with inarticulate speech and cognitive dysfunction. His muscle strength of bilateral lower limbs was reduced. The tendon reflexes of bilateral lower limbs were weakened, and bilateral Babinski signs were positive.

**FIGURE 3:**
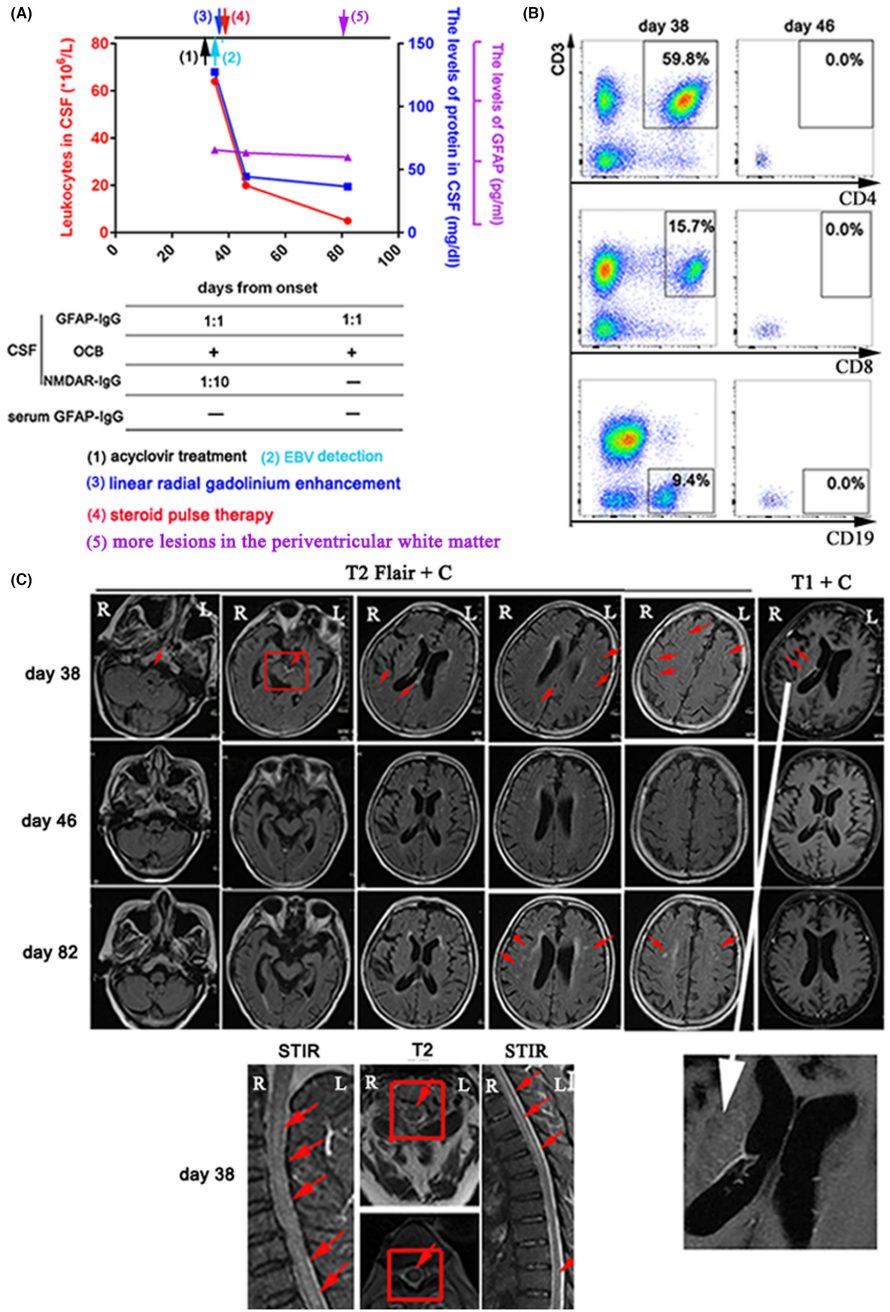
(A) CSF findings during EBV infection and follow‐up. Leukocytes in CSF: red circles and red lines, left y‐axis. The levels of protein in CSF: blue boxes and blue lines, right y‐axis. The levels of GFAP in CSF: purple triangle and purple lines, right y‐axis. −, negative; +, positive; OCB, oligoclonal bands. (B) The percentages of CD4^+^ T cells, CD8^+^ T cells, and B cells among lymphocytes in CSF were measured at different time points. (C) Gadolinium enhancement of brain MRI showed meningeal enhancement (including the meninge at midbrain interpeduncal fossa) and linear enhancement oriented radially to the ventricles (white arrow) on day 38. Spinal cord MRI showed longitudinally lesions involving the cervical and thoracic spinal cord. After hormonotherapy, brain MRI showed no meningeal enhancement on day 46. During follow‐up, image showed more T2/Flair‐hyperintense lesions in the periventricular white matter on day 82.

The clinical symptoms had mildly improved after acyclovir treatment. CSF analysis revealed elevated leukocytes (64*10^6^/L) with 70% monocytes and protein level of 127.5 mg/dL (Figure 3A). mNGS showed EBV DNA unique reads is six in CSF. GFAP‐IgG was positive in CSF (titer, 1:1) and negative in serum. Both NMDAR‐IgG (titer, 1:10) and OCB were positive in CSF. Importantly, CD4^+^ T cells, CD8^+^ T cells, and B cells were detected in CSF (Figure 3B). Brain MRI showed extensive meningeal enhancement and linear enhancement oriented radially to the ventricles on day 38. Spinal cord MRI showed longitudinally extensive lesions involving the cervical and thoracic segments (Figure 3C).

The patient was diagnosed as autoimmune GFAP astrocytopathy. The clinical symptoms were quickly improved after steroid pulse therapy. On day 82, GFAP‐IgG was also positive in CSF (titer, 1:1). The levels of GFAP (Figure 3A) and the percentages of CD4^+^ T cells, CD8^+^ T cells, and B cells (Figure 3B) in CSF were significantly reduced. However, on day 82, more lesions in the periventricular white matter were founded (Figure 3C). The patient received low dose of corticosteroid and mycophenolate mofetil. The clinical symptoms had no relapse after 5 months of clinical follow‐up.

## 4 DISCUSSION

GFAP uniquely presents in astrocytes of CNS, non‐myelinated Schwann cells and satellite glia cells of the peripheral nervous system, and enteric glia cells (EGC). GFAP participates in the formation of skeleton structure of astrocytes and blood–brain barrier (BBB), maintaining morphological stability of astrocytes.^1^, ^4^ Diagnosis of autoimmune GFAP astrocytopathy depends on the presence of GFAP‐IgG in CSF. GFAP is an intermediate filament protein. How GFAP‐IgG penetrates cell membrane to bind intracellular GFAP is still unclear. Autoimmune GFAP astrocytopathy can present after herpes simplex viral encephalitis, which indicates that virus might trigger immune response.^5^, ^6^ In this study, the clinical features of three autoimmune GFAP astrocytopathy patients with EBV infection are shown in Table 1. In case 1, both EBV and OCB were found in CSF but GFAP‐IgG on day 10 after onset. OCB can be directly caused by viral infection or immune response induced by viral infection. The clinical symptoms had mildly improved after antiviral treatment for 1 week. Both the clinical symptoms and brain MRI imaging had significantly improved after subsequent steroid pulse therapy. However, GFAP‐IgG in CSF was detected after 1 month later (titer, 1:10) accompanied by more periventricular lesions. It was also reported that a rare case of EBV encephalomyelitis with negative GFAP‐IgG at onset turned positive GFAP‐IgG after 15 days.^7^ The temporal sequence of EBV infection and GFAP‐IgG appearing indicated a causal mechanism. It was reported that autoimmune GFAP astrocytopathy could coexist with other antibodies, such as NMDAR‐IgG and AQP4‐IgG. Althought NMDAR‐IgG existed in the CSF of case 3, the clinical symptoms and the radial linear enhanced lesions perpendicular to the ventricle supported the diagnosis of autoimmune GFAP astrocytopathy. NMDAR‐IgG could be found in infection and autoimmune diseases of CNS as a concomitant antibody. In case 3, CD4^+^ T cells, CD8^+^ T cells, and B cells were detected in CSF. The patient had poor response to antiviral treatment and clinical symptom and pleocytosis were improved quickly after steroid pulse therapy, which also support the notion of EBV‐induced CNS autoimmune response. Variable numbers of EBV from one to thousands of sequence reads were reported by CSF mNGS in EBV encephalitis.^8^ Low sequence reads of EBV in CSF cannot eliminate the possibility of its active infection.

**TABLE 1:** Clinical features of autoimmune GFAP astrocytopathy patients.

No.	Age sex	Neuropathy symptoms	CSF				Imaging features	Coexisting autoimmune disease/cancer	Drug/immuotherapy response
			GFAP‐IgG	NMDAR‐IgG	OB	EBV DNA reads			
1	33 y F	Fever, headache, cognitive dysfunction, dysphoria, and limb weakness	1:10	−	+	2	Meningitis, linear enhancement oriented radially to the ventricles, and periventricular white matter lesions	No/no	Steroid + MM/improved
2	60 y M	Fever, abdominal pain, ventosity, limb shaking, limb weakness, cognitive dysfunction, and urine retention	1:100	−	+	1	Meningoencephalomyelitis, linear enhancement oriented radially to the ventricles, and periventricular white matter lesions	No/no	Steroid + MM/improved
3	71 y M	Fever, urine retention, occasional ventosity, loss of appetite, cognitive dysfunction, hand tremors, and limb weakness	1:1	1:10	+	6	Meningoencephalomyelitis, linear enhancement oriented radially to the ventricles, and periventricular white matter lesions	No/no	Steroid + MM/improved

Abbreviations: CSF, cerebrospinal fluid; EBV, Epstein–Barr virus; F, female; GFAP, glial fibrillary acidic protein; M, male; MM, mycophenolate mofetil; NMDAR, N‐methyl‐D‐aspartate receptor; OB, oligoclonal bands.

There are some potential pathogenic mechanisms of EBV that may lead to CNS disorders: ① The virus directly invades nervous system. EBV may enter the brain via normal B cell trafficking or through infection of brain microvasculature endothelial cells.^3^ EBV infection of the microvasculature endothelial cells and B cells may be the source of neurotoxicity through the release of inflammatory cytokines (IL‐6, TNF‐α, and IL‐1), free radicals, and viral proteins, which in turn lead to neuronal damage^3^ and may further result in perivascular inflammation. ② T cell‐mediated responses to infected cells in the brain may lead to bystander damage. EBV‐specific cytotoxic CD8^+^ T cell responses is regarded as the main determinant of CNS tissue damage.^9^ CD8^+^ T cell might be an important pathological and diagnostic feature in autoimmune GFAP astrocytopathy.^10^ ③ Infection‐mediated immune response. EBV may share a common antigen with nervous system, which can produce autoimmune T lymphocytes and antibodies to autoantigens.^11^ EBV‐infected autoreactive B cells accumulate in the CNS where they can produce pathogenic antibodies and provide costimulatory survival signals to autoreactive T cells.^12^ ④ Reactivation of latent infection. In the latent phase, memory B cells are the main reservoir of EBV. EBV is prevented from reactivating through efficient cytotoxic T cells. EBV reactivation has been shown to occur in a variety of autoimmune diseases, including multiple sclerosis, systemic lupus erythematosus, and rheumatoid arthritis. Reactivation of EBV in brain microvasculature endothelial cells and B cells could upregulate cytokines, chemokines, and adhesion molecules that would induce a local breach in the BBB and attract autoreactive lymphocytes into the brain.^13^ The pathological features revealed marked inflammatory responses in perivascular regions, including CD4^+^ T cells, CD8^+^ T cells, B cells, plasma cells, macrophages, and neutrophils in autoimmune GFAP astrocytopathy.^14^, ^15^

EGC is an important component of enteric nervous system (ENS) that reside within the smooth muscle wall, submucosa, and lamina propria. EGC shares morphological features and antigenic markers with the astrocytes of the CNS.^16^ EGC can interact with lymphocytes, respond actively to inflammation, and become activated as antigen‐presenting cells attracting immune cells to the ENS.^17^ EGC plays important roles in gut homeostasis through the release of various trophic factors and contribute to the integrity of the epithelial barrier, gut motility.^18^ Notably, CD8^+^ T cell‐mediated cytotoxic autoimmunity might involve in EGC abnormalities, which were associated with massive intestinal inflammation.^19^ In patient 2, GFAP‐IgG in serum could partly colocalize in small intestine of rat. We speculated that the symptom of abdominal pain and ventosity might be related to the disruption of EGC.

Based on our data, we propose that EBV infection in brain microvasculature endothelial cells and B cells could lead to not only neuronal damage but also a breakdown of the BBB, which can attract autoreactive lymphocytes into the CNS and further result in perivascular inflammation. In our patients, typical imaging findings include the gadolinium enhancement of linear perivascular radial perpendicular to the ventricle, meningeal enhancement (especially in midbrain interpeduncal fossa), longitudinally extensive lesions involving spindle cords, and more T2/Flair‐hyperintense lesions in the periventricular white matter at late stage. The gadolinium enhancement of linear perivascular radial perpendicular to the ventricle and more T2/Flair‐hyperintense lesions in the periventricular white matter at late stage may be related to the perivascular inflammation induced by EBV and higher expression of GFAP in white matter compared to gray matter astrocytes.^20^ According to our data, we recommend maintaining vigilance against cases of suspected viral encephalitis with poor response to antiviral therapy, and the detection of GFAP‐IgG and EBV may facilitate the early diagnosis in these patients.

## AUTHOR CONTRIBUTIONS

RSD, BY, and XLL contributed to the study concept and design. RSD, XLL, LKL, JYW, CLY, XLZ, PZ, BL, and YBL participated in the data the data acquisition and analysis. RSD, XLL, LKL, JYW, and CLY contributed to the drafting of the manuscript. XLL, CLY, and ZXZ contributed to drafting the text and preparing the figures. All authors read and approved the final manuscript.

## FUNDING INFORMATION

This work was supported by grants from Academic promotion program of Shandong First Medical University (2019QL013), National Natural Science Foundation of China (81601040, 81303013), and Cultivation Fund for the First Affiliated Hospital of Shandong First Medical University (QYPY2020NSFC0610).

## CONFLICT OF INTEREST STATEMENT

The authors declare that they have no competing interests.

## Data Availability

The raw data supporting the conclusions of this article will be made available by the authors, without undue reservation.
